# Motor planning stage of gait initiation: effects of aging, Parkinson’s disease, and associations with cognitive function

**DOI:** 10.1007/s00221-025-07151-3

**Published:** 2025-08-28

**Authors:** Galia Shaham, Irina Galperin, Amit Salomon, Eran Gazit, Aron S. Buchman, Nir Giladi, James K. Richardson, Jeffrey M. Hausdorff

**Affiliations:** 1 Center for the Study of Movement, Cognition and Mobility, Neurological Institute, Tel Aviv Sourasky Medical Center, 6 Weizmann Street, Tel Aviv, Israel; 2 Sagol School of Neuroscience, Tel Aviv University, Tel Aviv, Israel; 3 Gray Faculty of Medical and Health Sciences, Tel Aviv University, Tel Aviv, Israel; 4 Department of Physical Medicine and Rehabilitation, University of Michigan, Ann Arbor, MI USA; 5 Rush Alzheimer’s Disease Center, Rush University Medical Center, Chicago, IL USA; 6 Department of Physical Therapy, Gray Faculty of Medical and Health Sciences, Tel Aviv University, Tel Aviv, Israel

**Keywords:** Parkinson’s disease, Elderly, Gait initiation, Reaction time, Response inhibition, Cognition, Aging

## Abstract

The response to a request to walk involves a motor planning phase followed by an execution phase. The initial phase of gait initiation, specifically the time to anticipatory postural adjustment (APA), can be viewed as a form of reaction time. However, it is not clear how to characterize the cognitive processes involved in this stage. To address this question, *time-to-APA*, simple and complex upper limb visuomotor reaction time (SRT, CRT), cognitive, and motor performance were evaluated in 27 people with Parkinson’s disease (PD), 31 older adults (OA), and 34 young adults (YA). Our results showed that *time-to-APA* was significantly longer than SRT in all three groups (*p* < 0.001), indicating a more complex cognitive process. In YA, *time-to-APA* was significantly shorter than CRT (*p* < 0.001). In the OA and PD, *time-to-APA* was not significantly different from CRT. Mixed-effects analysis showed significant time (*p* < 0.001), group (*p* = 0.037), and group × time interaction effects (*p* = 0.002). Among all subjects, *time-to-APA*, but not APA duration, was associated with the Color-Trails Test (part B: r_s_ = 0.406, *p* < 0.001). In PD, APA duration was correlated with MDS-UPDRS-part 3 (motor) scores (r_s_ = 0.535, *p* = 0.004), but *time-to-APA* was not (*p* = 0.892). These findings suggest that *time-to-APA* is a cognitive process that is more complex than an SRT task and shares properties of a CRT task, especially among older adults and people with PD. In PD, this initial movement planning stage is not related to motor impairment, in contrast to APA duration. Further research is necessary to identify the factors underlying this initial stage of gait initiation.

**Supplementary Information:**

The online version contains supplementary material available at 10.1007/s00221-025-07151-3.

## Introduction

Gait initiation (GI) refers to the transition from quiet standing to steady-state walking and can be divided into several stages (Cohen et al. 2017; Gazit et al. 2020). The first phase involves a volitional decision to start walking, followed by a subconscious movement planning stage before actual movement occurs. This initial planning stage, known as time to anticipatory postural adjustment (*time to APA*), precedes the APA phase, which involves postural shifts necessary for stable walking. During the APA phase, significant weight shifts help stabilize the body, prevent falls before taking the first step, and unload the swing leg, preparing it for the first step.

Previous studies indicate that APA characteristics—magnitude, shape, and duration—affect subsequent gait patterns (Filho et al. 2021; Gazit et al. 2020). These APA features are altered by aging (Duarte et al. 2022) and neurological diseases such as Parkinson’s disease (PD) (Delval et al. 2014a; Palmisano et al. 2020, 2022; Schlenstedt et al. 2017; Waele et al. 2024; Rogers et al. 2011). However, while APA characteristics have been studied extensively, the cognitive mechanisms underlying *time to APA* remain unclear, particularly what cognitive resources are involved and how they differ between young adults, older adults, and individuals with PD.

An efficient transition from standing to walking is critical for functional mobility (Rocchi et al. 2006). A prolonged *time to APA* may indicate difficulties in motor planning and cognitive control (Filho et al. 2021; Schlenstedt et al. 2017). Because *time to APA* represents the interval between the decision to move and the onset of postural adjustment, it can be viewed as a form of reaction time. A key question is whether *time to APA* resembles a simple reaction time (SRT) task, requiring minimal cognitive processing, or a complex reaction time (CRT) task that engages executive function and response inhibition.

One perspective suggests that *time to APA* is largely automatic, requiring minimal cognitive involvement. From this viewpoint, it would be similar to an SRT task, which reflects the rapid execution of a simple motor response. Alternatively, *time to APA* might require task switching, inhibition of prepotent motor responses, and higher-order cognitive control—features characteristic of a CRT task. To explore this, we compared *time to APA* with SRT and CRT using an upper extremity task, the ReacStick (Eckner et al. 2010; Richardson et al. 2017).

In particular, this study aimed to: a) Determine whether *time to APA* is similar in duration to an upper extremity SRT task or CRT task among healthy young adults. b) Examine whether *time to APA* is affected by aging and PD, given that both are associated with motor deficits and cognitive impairments. And c) Assess the cognitive and motor correlates of *time to APA*. As an exploratory objective, we also examined whether *time to APA* and APA duration are differentially associated with PD motor symptoms.

## Methods

### Study participants

This cross-sectional study was conducted at the Center for the Study of Movement, Cognition, and Mobility (CMCM), Neurological Institute, Tel Aviv Sourasky Medical Center, Tel Aviv, Israel, and approved by the local ethics Helsinki committee. Participants were enrolled after providing informed written consent. For all subjects, the inclusion criteria included the ability to walk unassisted and adequate vision capabilities (normal or corrected to normal vision as defined by the ability to read and distinguish between dissimilar colors). Young adults (YA) were included if they were between 18 and 39 years old. The older adults (OA) and those with Parkinson’s disease (PD) were included if they were between 50 and 85 years old and Hoehn and Yahr stage between 1 and 3 (inclusive). The patients with PD received a diagnosis of PD from a neurologist specializing in movement disorders. The subjects with PD were evaluated in their self-declared “On medication” state. Exclusion criteria for all subjects included a diagnosis of an orthopedic or other neurological condition likely to affect the results of the research, in addition to any cardiovascular or musculoskeletal impairment that prohibited them from walking 10 m without a walking aid. Participants scoring below 22 on the Montreal Cognitive Assessment (MoCA) were also excluded. Patients with freezing of gait, as determined via visual observation or medical history, were excluded as well. As described further below, we assessed mobility, reaction time performance, cognitive performance, and demographic and clinical characteristics. In addition, PD disease characteristics were evaluated using the MDS-Unified Parkinson’s Disease Rating Scale (MDS-UPDRS) (Goetz et al. 2008).

### Evaluation of mobility and gait

Gait initiation was tested by asking the subject to start walking from a standing position after hearing a tone (Gazit et al. 2020). Subjects were instructed to stand in a neutral comfort position with feet hip-width apart, to start walking as soon as possible after hearing a pre-specified tone, and to walk at their normal speed.

The tone was randomly initiated within a 5-s range, and both the subjects and the examiner were blinded to its exact timing. The choice of a random delay was used to minimize the possibility that subjects would learn to predict when the start signal would occur. Gait initiation testing was repeated three times under random conditions. Subjects were not instructed to initiate gait with a particular leg; it was their choice in each trial, i.e., it was a randomly chosen leg. The duration of each stage was averaged over the three trials. The Zeno Walkway (Protokinetics LLC, Havertown, PA) was used to measure APA and gait initiation (Vallabhajosula et al. 2019). The time from the auditory cue until the start of anticipatory postural adjustment (APA) was defined as *time to APA* and determined as previously described (Gazit et al. 2020). As described previously (Gazit et al. 2020), to define the time of the APA, we utilized the MATLAB function 'findchangepts,' which detects significant changes or transitions in time-series data. We also verified that the identified point signifies the transition from quiet standing to the beginning of movement. Thus, using the definitions previously described by Vallabhajosula et al. (2019) and Gazit et al. (2020), *time to APA* was defined as the time from the start of the auditory cue to the start of the APA; the end of the APA was identified by the start of toe-off and the start of the APA was identified by searching from the end of the APA to a point where the accelerometer signals return to baseline values (i.e., end of quiet standing and the beginning of weight shifting). That point was defined as the start of APA.

APA duration was also determined to contrast this movement implementation time to the movement “planning time”, as previously described (Gazit et al. 2020). In addition to *time to APA*, we also determined gait speed and step length using the Zeno walkway software. We excluded the first two steps and the last step from each of the three trials to calculate the average of each gait.

The mobility evaluation included the assessment of gait initiation, gait, the Timed Up and Go test during usual walking (single task) and during a dual task (Podsiadlo and Richardson 1991), and the Four Square Step (Dite and Temple 2002). The Timed Up and Go assessments included two conditions: usual and dual-task conditions (verbalized serial subtraction of 3 s).

### Upper extremity reaction time tests

#### SRT

The ‘ReacStick’ was used to evaluate upper limb extremity reaction time (RT) under two conditions (Eckner et al. 2010, 2012; Ellmers et al. 2021; Richardson et al. 2017). The first condition is simple RT (SRT), where the subject grasps a falling, ruler-like device as quickly as possible upon random release of the ‘ReacStick’. SRT was analyzed as a measure of movement initiation.

#### CRT

The second condition is complex RT (CRT), using a go/no go paradigm; the subjects were instructed to grasp the device only during the random 50% of trials when green lights on the device were illuminated. Otherwise, the instruction was to let the stick drop to the floor, which requires short latency (< 390 ms when released from 31 cm) inhibition (Richardson et al. 2017). The timing of the release of the ‘ReacStick’ was also random. Given the pre-potent inclination to quickly catch the falling device, even when the lights did not illuminate, inhibition was determined by CRT Off Accuracy (the % of the trials in which the stick was correctly not grasped, when the lights on the stick were not illuminated), the degree of prolongation of CRT on appropriately caught with the light on trials as compared to SRT (CRT—SRT), and Total Inhibition, the sum of two inhibition measures: [light Off Accuracy plus (CRT—SRT)].

#### Cognitive tests

To further characterize the study participants and to evaluate the association between *time to APA* and motor and cognitive function, several tests were included in addition to the MoCA test. The Color Trail Test (CTT) A and B were administered to assess visual scanning, and attention (D’Elia et al., 1996). During the CTT-A, the subject was instructed to connect the numbers 1–25 in ascending order without lifting the hand from the page. During CTT-B, subjects also needed to alternatively connect between two symbol colors. The tests were timed, and the CTT factor score was calculated as the difference between the duration of the two parts, i.e., CTT-B minus CTT-A. This CTT score was used to indicate cognitive efficiency and executive function (Ho et al. 2018).

### Statistical analyses

Statistical analyses were conducted using Matlab (R2021b) and Prism 9.5.1 (2023). The Shapiro–Wilk test was used to test for normality. Within and between-group effects were evaluated using Kruskal–Wallis tests for continuous measures. Gender distribution was compared using chi-squared tests. Spearman’s correlation coefficients quantified relationships between measures; partial correlation analyses were used to examine if correlations remained significant after controlling for age, sex, and weight. Multiple comparisons were treated via Bonferroni corrections. Bonferroni corrections were applied according to the number of comparisons. The Friedman test was used to identify outliers using ROUT (Q = 1%); outliers were removed from the analysis. To compare SRT, CRT, and *time to APA* across and within groups*,* mixed-effects analysis (REML) examined time, group differences, and time X group interaction as fixed effects, while subjects were considered as random effects.

## Results

### Demographic and cognitive characteristics

Ninety-five subjects were recruited, and three were later excluded (one older adult and one subject with PD with a MoCA test score < 22, and another subject with PD who entered the off-medication state during testing). Thus, 92 subjects were included in the final analysis: 34 healthy young adults, 31 healthy older adults, and 27 older adults with PD. Subject characteristics are summarized in Table 1. The gender distribution did not significantly differ across the three groups (χ^2^ = 0.69, *p* = 0.705). Height and weight also did not significantly differ among the three groups (*p* > 0.11). Age, years of education, gait speed, and step length were significantly different among the three groups (*p* < 0.004). There were no significant differences between the older adults and people with PD for any of the characteristics (*p* > 0.176). As expected, the young adults had higher values of gait speed and step length, compared to the two other groups.

**Table 1:** Subject demographics, motor and cognitive performance

	Young adults (n = 34)	Older adults (n = 31)	People with PD (n = 27)
Age (years)	28.14 ± 5.56*	66.90 ± 6.16	67.40 ± 8.37**
Gender (% female)	47.1%	54.8%	44.4%
Education (years)	16.94 ± 2.81	15.81 ± 3.04	14.52 ± 2.58**
Height (cm)	169.84 ± 10.52	167.06 ± 8.88	168.14 ± 9.30
Body mass (kg)	68.28 ± 13.44	75.41 ± 11.99	73.37 ± 10.74
*Gait Initiation*			
Time to APA (msec)	182.2 ± 21.5	208.6 ± 49.8	219.9 ± 52.21**
APA duration (msec)	484.92 ± 121.02	520.47 ± 129.89	594.40 ± 147.63**
*Gait Measures*			
Gait speed (cm/s)	156.27 ± 28.60*	130.16 ± 25.04	123.61 ± 25.15**
Step length (cm)	76.80 ± 7.43*	69.03 ± 9.32	65.22 ± 9.73**
*Mobility*			
Timed Up and Go (sec)	8.29 ± 0.93	8.80 ± 1.98	9.74 ± 2.06**
Dual Task Timed Up and Go (sec)	8.88 ± 1.25	9.88 ± 2.73	11.23 ± 3.11**
Four Square Step Test (sec)	7.93 ± 1.37*	10.27 ± 2.44	11.61 ± 3.70**
*Cognitive Function*			
MoCA (0–30)	28.88 ± 1.25*	26.74 ± 2.05	26.85 ± 1.73**
CTT-A (sec)	27.21 ± 8.73*	55.16 ± 17.81	68.41 ± 39.67**
CTT-B (sec)	55.08 ± 15.07*	118.01 ± 41.25	126.68 ± 45.63**
CTT-B—CTT-A (sec)	27.87 ± 13.33*	62.86 ± 32.43	58.27 ± 34.84**
*ReacStick measures*			
CRT-SRT (msec)	62.24 ± 31.90	44.99 ± 34.13	30.70 ± 24.27**
Total Inhibition score	140.92 ± 38.97*	89.77 ± 47.40	76.42 ± 38.36**
*PD specific measures*			
Total MDS-UPDRS	NA	NA	46.1 ± 23.4
MDS-UPDRS part 3	NA	NA	24.7 ± 16.0
Hoehn and Yahr stage	NA	NA	1.7 ± 0.6 (range:1–3, Median: 2)
Years since diagnosis	NA	NA	6.0 ± 4.6
Levodopa Equivalent Daily Dose (LEDD) (mg)	NA	NA	464.2 ± 359.0

^*^significant difference (*p* < 0.05/3) between young adults and older adults after Bonferroni correction; **significant difference (*p* < 0.05/3) between young adults and people with PD after Bonferroni correction. ***significant difference (*p* < 0.05/3) between older adults and people with PD after Bonferroni correction. Entries are mean ± SD and percentage for gender. NA: not applicable

### Reaction time evaluation

**Fig. 1:**
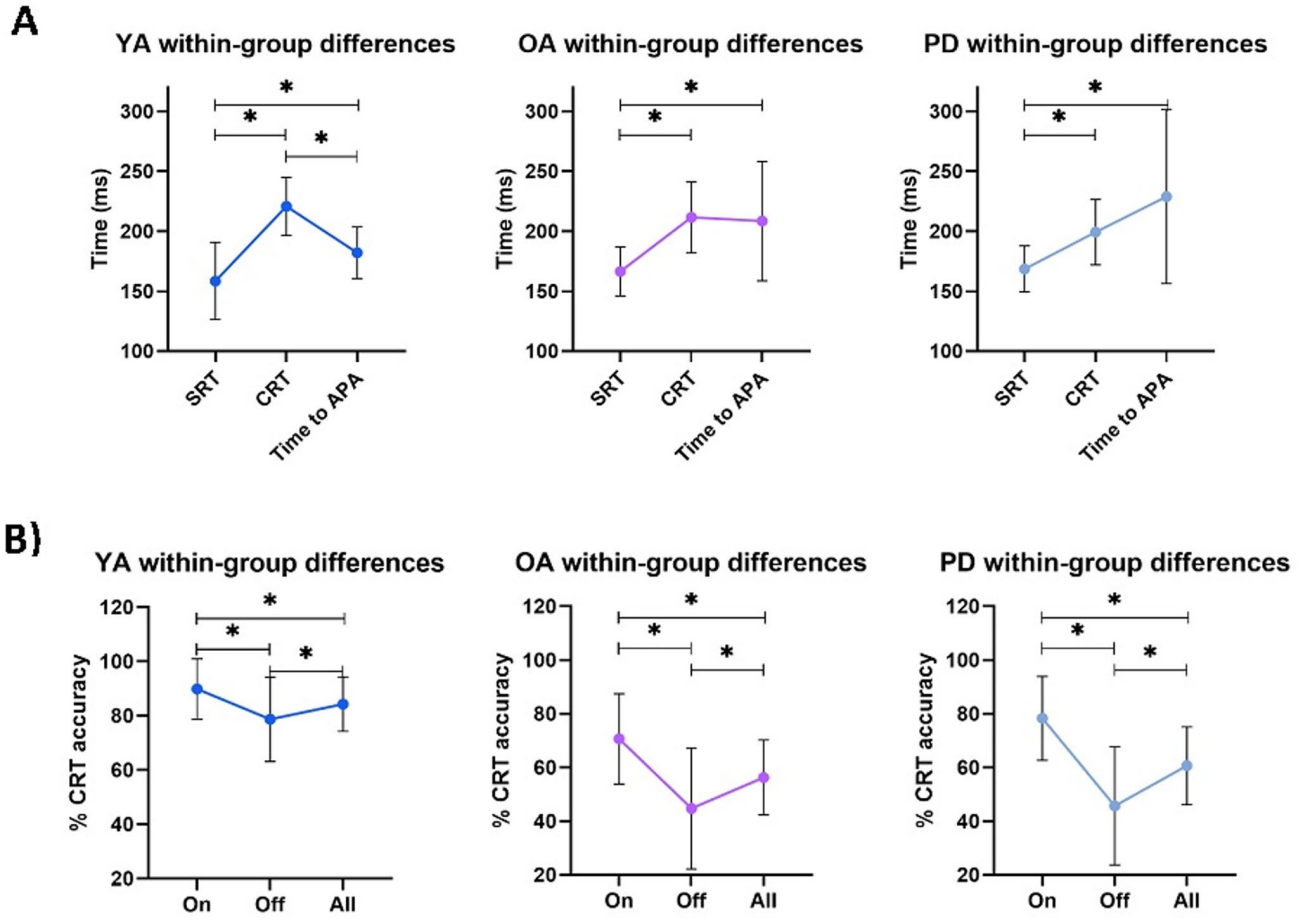
Within-group differences in reaction time (**A**) and CRT accuracy (**B**). See also the supplementary material. *Significant after a Bonferroni correction

Within-group comparisons revealed that *time to APA* was significantly longer than SRT (*p* < 0.001) in all three groups. As expected, CRT was also longer than SRT in all three groups (*p* < 0.001). In addition, in the young adults, *time to APA* was significantly shorter than CRT (*p* < 0.001). In the older adults and older adults with PD, *time to APA* was not significantly different from CRT duration (see Fig. 1A and supplementary information Table 1).

Mixed-effects analysis evaluating SRT, CRT, and *time to APA* showed significant effects for time (*p* < 0.001), group (*p* = 0.037), and the group X time interaction (*p* = 0.002). As summarized in Table 2 in the supplementary information, post hoc analysis revealed that young adults had significantly shorter SRT and *time to APA* values (*p* < 0.002), and longer CRT (*p* < 0.001), compared to people with PD (please also see Tables 2 and 3 in the supplementary material). Other across-group comparisons of the task durations were not significant.

**Table 2:** Correlations between: (A) Time to APA and ReacStick reaction times and accuracy parameters, (B) Time to APA and cognitive function, (C) Time to APA and gait and mobility measures and (D) Time to APA and mobilityy

Time to APA correlation with:	All Subjects	Young Adults	Older Adults	People with PD
(A)				
SRT	0.120 (0.255)	0.075 (0.674)	0.035 (0.851)	− 0.098 (0.627)
CRT	− 0.103 (0.329)	0.190 (0.283)	− 0.144 (0.439)	− 0.130 (0.518)
CRT—SRT	− 0.165 (0.116)	0.129 (0.466)	− 0.139 (0.454)	0.083 (0.679)
CRT off accuracy	− 0.248 (0.017)	− 0.041 (0.819)	− 0.111 (0.551)	− 0.167 (0.404)
Total Inhibition score	− 0.264 (0.011)	− 0.029 (0.872)	− 0.211 (0.254)	− 0.078 (0.701)
(B)				
MoCA	− 0.263 (0.011)	− 0.119 (0.502)	− 0.081 (0.666)	− 0.187 (0.350)
CTT-A	0.418 (*p* < 0.001)* ×	0.197 (0.265)	0.433 (0.015)	0.3380 (0.083)
CTT-B	0.406 (*p* < 0.001)*	0.352 (0.041)	0.342 (0.060)	0.184 (0.357)
CTT-B—CTT-A	0.288 (0.005)*	0.374 (0.030)	0.133 (0.474)	0.036 (0.857)
(C)				
Gait speed	− 0.383 (0.001)* ×	0.016 (0.929)	− 0.317 (0.088)	− 0.362 (0.064)
Step length	− 0.249 (0.019)*	0.309 (0.085)	− 0.108 (0.567)	− 0.410 (0.034)
(D)				
Timed Up and Go	0.298 (0.004)* ×	0.138 (0.438)	− 0.018 (0.924)	0.654 (*p* < 0.001)* ×
Dual Task Timed Up and Go	0.465 (*p* < 0.001)* ×	0.464 (0.006)* ×	0.386 (0.035)	0.426 (0.023)
Four Square Step Test	0.339 (0.001)*	− 0.211 (0.231)	0.423 (0.020)	0.342 (0.088)

Results shown are Spearman correlation coefficient (rho, *p*-value); *Significant after a Bonferroni correction. × Remains significant after adjusting for age, sex, and weight (and after a Bonferroni correction). MoCA: Montreal Cognitive Assessment. CTT: Color Trails Test. **Refers to the condition during which the lights on the stick were not illuminated

**Table 3:** Correlations between Time to APA, APA duration, and cognitive function measures (among all subjects)

	Time to APA	APA Duration
Montreal Cognitive Assessment	0.263 (*p* = 0.011)	0.180 (*p* = 0.116)
Total Inhibition score	0.264 (*p* = 0.011)	− 0.216 (*p* = 0.038)
CTT-A	0.418 (*p* < 0.001)* ×	0.190 (*p* = 0.096)
CTT-B	0.406 (*p* < 0.001)*	0.210 (*p* = 0.065)
CTT-B–CTT-A	0.288 (*p* = 0.005)*	0.121 (*p* = 0.291)

Results shown are Spearman correlation coefficient (rho, *p*-value); *Significant after a Bonferroni correction (*p* < 0.05/5). × Remains significant after adjusting for age, sex and weight (and after a Bonferroni correction)

We assessed the CRT response accuracy for Go signals and No-go signals, as part of the ‘ReacStick’. Post hoc comparisons revealed that CRT Off accuracy (when the lights on the stick were not illuminated) was significantly lower in the older adults (*p* < 0.001) and people with PD (*p* < 0.026), compared to the young adults (Supplementary Information Tables 2 and 3). Of note, CRT-SRT was positively correlated with off accuracy among all subjects (rs = 0.512, *p* < 0.001), indicating that better Off accuracy (when the lights on the stick were not illuminated) waas related to CRT prolongation. CRT prolongation and Total Inhibition scores were longest in the young adults, smaller in the older adults, and reduced further in people with PD (recall Table 1). However, there were no significant differences between the older adults and people with PD on all three accuracy measures (*p* > 0.90). The *p*-values of all comparisons are detailed in supplementary information Table 2 (see also supplementary information Table 3).

#### Time to APA correlations

Among all participants, *time to APA* tended to be inversely related to the Total Inhibition score (Table 2A), such that diminished Total Inhibition was mildly associated with prolonged *time to APA*. *Time to APA* was also significantly correlated with performance on the conventional cognitive tests: MoCA, CTT-A, CTT-B, and CTT-B-A, as summarized in Table 2B. In other words, a longer *time to APA* was associated with worse cognitive performance. Table 2C and D show the correlations between *time to APA* and gait and mobility measures. *Time to APA* was significantly correlated with both gait speed and step length. Here too, longer *time to APA* values were related to worse performance. These correlations were not significant in the per-group correlation analysis. The correlation between *time to APA* and Timed Up and Go times was driven primarily by the PD group. Among people with PD, a long *time to APA* was positively correlated with a longer time to complete the Time Up and Go. *Time to APA* was also associated with performance on the Four Square Step Test and dual-task Timed Up and Go; the correlation between *time to APA* and Timed Up and Go Dual-Task was driven primarily by the young adults. Scatter plots of these relationships are shown in Supplementary information Fig. 1.

Table 3 contrasts the associations between *time to APA* and APA duration and cognitive function among all subjects. *Time to APA* was associated with all cognitive measures, while APA duration was not associated with any of these cognitive measures.

Among the individuals with PD, *time to APA* and APA duration were moderately correlated with each other (rs = 0.351, *p* = 0.002). As shown in Table 4, among people with PD, *time to APA* was not significantly (*p* > 0.9) correlated with disease duration, MDS-UPDRS total, MDS-UPDRS part 3, or LEDD. In contrast, APA duration was associated with MDS-UPDRS part 3 (rs = 0.535, *p* = 0.004) but not with disease duration or LEDD.

**Table 4:** Correlations between Time to APA, APA duration, and Parkinson's disease characteristics measures (among people with PD)

	Time to APA	APA Duration
Disease Duration	− 0.020 (*p* = 0.921)	0.109 (*p* = 0.588)
MDS-UPDRS total	0.026 (*p* = 0.898)	0.431 (*p* = 0.025)
MDS-UPDRS part 3	− 0.028 (*p* = 0.892)	0.535 (*p* = 0.004)* ×
LEDD	0.328 (*p* = 0.095)	0.139 (*p* = 0.488)

Results shown are Spearman correlation coefficient (rho, *p*-value); *Significant after a Bonferroni correction (*p* < 0.05/4). × Remains significant after adjusting for age, sex, and weight (and after a Bonferroni correction)

*Time to APA* was correlated with age in the YA group (r_s_ = − 0.58 *p* = 0.0005) and among all subjects (r_s_ = 0.28 *p* = 0.0066). When looking at the PD and OA as one group, there was a trend for an association with age (r_s_ = 0.30 *p* = 0.023), However, among the PD and among the OA, *time to APA* was not significantly associated with age. *Time to APA* was not significantly associated with weight or sex in any group or grouping.

## Discussion

This study investigated *time to APA* as a measure of motor planning and its relationship to cognitive and motor function across young adults, older adults, and individuals with PD. The results suggest that *time to APA* is not merely a simple motor response but also relies on executive function and response inhibition, particularly in older adults and individuals with PD.

### Time to APA as a cognitive process

*Time to APA* was not associated with upper extremity SRT. Since both *time to APA* and upper extremity SRT are, putatively, forms of a reaction timed test, one might have anticipated observing correlations between these two tests; but we did not (recall Table 2A; *p* > 0.25). SRT is a simple visuomotor task most likely generated by the premotor cortex which activates the basal ganglia and inhibits the globus pallidus, leading to excitation of the motor cortex (Aron et al. 2007). Notably, upper limb SRT does not appear to involve a large degree of prefrontal cortical resources, possibly explaining why no association was found between it and *time to APA.* An additional factor that may explain the lack of an association is that the SRT test includes the beginning of a motor task, while *time to APA* does not.

We found, instead, that *time to APA* was longer than SRT in all groups, reinforcing the idea that it is not a simple motor response. Interestingly, among young adults, *time to APA* was shorter than CRT duration. One interpretation of this finding is that *time to APA* is a relatively automatic task in healthy young adults, perhaps more analogous to a SRT than a CRT, involving minimal cognitive resources, especially as compared to the older adults and people with PD. In the young adults, time to APA was, nonetheless, longer than SRT, perhaps due to differences related to upper versus lower function or specifics of the two tasks (Pfister et al. 2014).

In contrast, among the older adults and individuals with PD, *time to APA* was comparable to CRT. Some have posited that the CRT reflects short latency inhibition performance (Antonelli et al. 2011; Yang et al. 2018). The present findings would suggest that in aging and PD, *time to APA* increasingly resembles a cognitively demanding task rather than an automatic motor reaction and that *time to APA* engages higher-level planning and decision-making mechanisms.

Furthermore, *time to APA* was significantly associated with performance on cognitive tests, including the MoCA test and the Color Trails Test (CTT). Longer *time to APA* correlated with worse cognitive performance, supporting the idea that this phase of gait initiation requires executive function, rather than merely reflecting motor readiness. In contrast, APA duration was not related to cognitive measures but was associated with PD motor severity, reinforcing its role as a marker of motor impairment rather than cognitive processing.

As shown in Table 2, the correlations between *time to APA,* on the one hand, and cognitive function, gait, and mobility, on the other hand, were strongest when examining all subjects as one group and weaker or not significant when examining correlations within each group. For example, *time to APA* was significantly (*p* < 0.001) correlated with CTT-A among all subjects, but this correlation was not observed in the young adults and was less strong in the older adults (*p* = 0.015) and the people with PD (only a trend). One interpretation of this and related findings (e.g., recall Table 2C and D) is that the association between CTT-A and *time to APA* was driven by the differences across groups, rather than within homogeneous groups individually. This underscores the importance of considering population heterogeneity. Another, complementary possibility that could also contribute to these explanations relates to the sample size. By definition, the sample size was largest when we evaluated all subjects as one group and much smaller when studying within-group correlations. Future work is needed to test these different possibilities.

### Motor planning and response inhibition

Our findings align with prior research on motor initiation deficits in PD and aging (Antonelli et al. 2011; Schlenstedt et al. 2017). Individuals with PD and, to a lesser extent, older adults showed lower Total Inhibition scores and a smaller CRT-SRT cost, suggesting difficulties in suppressing prepotent motor responses. These deficits may contribute to prolonged *time to APA*, as successful gait initiation likely requires inhibitory control to transition from standing to walking. Indeed, people with PD, and to some extent older adults, have deficiencies in movement initiation and motor impulsivity (Antonelli et al. 2011; Doridam et al. 2019; Levin et al. 2014; Wylie et al. 2012). Those impairments may be associated with a longer *time to APA* (Gazit et al. 2020; Schlenstedt et al. 2017), perhaps due to inefficient ability to initiate desired motor responses and inhibition of the prepotent motor response (Cohen et al. 2017).

### Bradyphrenia and the group × time effect

A key finding was the significant group × time interaction, which indicates that aging and PD do not uniformly affect all reaction times. If bradyphrenia (slowed cognitive processing in PD) were the sole explanation, we would expect parallel increases in *time to APA*, SRT, and CRT across groups. However, we observed that *time to APA* was disproportionately prolonged in older adults and individuals with PD, suggesting distinct underlying mechanisms. The greater reliance on frontal cortical resources and executive function may explain why *time to APA* is particularly affected in these populations, as suggested previously (Richard et al; 2017). Studies of other aspects of gait have also reported an increased reliance on executive function and frontal cortical areas in aging and PD, putatively to compensate for impairments and deficits in motor function (Bayot et al. 2023; Richard et al; 2017; Yogev-Seligmann et al. 2008; Kahya et al. 2019). Nonetheless, the cross-sectional nature of this study limits interpretations regarding cause and effect.

Recent studies suggest that other factors may also contribute to *time to APA* nulli. For example, De Waele et al. (2024) reported that *time to APA* was related to weight and that age played a significant role in the second phase of gait initiation in people with PD. In contrast, in a study of unmedicated people with PD, Palmisano et al. (2020) found that changes in the APA related to the “imbalance phase” (initial APA phase) were correlated with the dopaminergic innervation of the putamen and substantially improved with levodopa but were not influenced by anthropometric parameters. Some authors also suggest differences in APA among patients with PD related to the presence or absence of freezing of gait (Palmisano et al 2020, 2022; Lencioni et al. 2024; de Lima-Pardini et al. 2020; Taximaimaiti and Wang 2021). On that note, it is also interesting to consider the suggestion that APA errors (i.e., alterations in timing, amplitude, sequence, and the need for multiple APAs) are larger in patients with PD and freezing of gait (Bayot et al. 2023). In the present study, patients with PD and freezing were not included. Follow-up studies directly comparing time to APA and potential explanatory factors (Gerard et al. 2024; Jacobs et al. 2009; Silva-Batista et al 2024) in early and more advanced PD and in PD patients with and without freezing of gait can help shed light on these open questions and the role of these additional potential mechanisms.

### Clinical and functional implications

A shorter *time to APA* was associated with better gait and mobility performance, including faster gait speed and longer step length. This suggests that efficient motor planning contributes to smoother gait transitions and overall mobility. Importantly, these relationships were strongest in individuals with PD, where longer *time to APA* correlated with slower performance on the Timed Up and Go test. Given that impaired gait initiation is a predictor of falls, *time to APA* may serve as a valuable marker for fall risk and functional decline in PD and aging, consistent with previous suggestions (Hu et al. 2024).

### Limitations and future directions

This study has several limitations. First, we assessed cued gait initiation using auditory stimuli rather than self-initiated movement. The basal ganglia, which are affected in PD, may be more involved in self-paced movement than in externally triggered tasks (Toyomura et al. 2012). Moreover, external cues are known to facilitate the generation of rhythmical stepping and to improve step preparation in people with PD (Toyomura et al. 2012; Delval et al. 2014b; Russo et al. 2022). The cued gait initiation allowed us to extract the specific planning stage of gait initiation, before any movement occurred, but it may interfere, to some degree, with the interpretation of study results. This may contribute to the lack of differences between the older adults and adults with PD in gait initiation stage duration and reaction time duration. Future research should examine whether similar patterns emerge in self-initiated gait.

Second, the number of trials per participant was relatively low (three repetitions), which may have reduced the reliability of *time to APA* measurements. Some suggest that at least ten trials may be necessary for robust gait initiation analysis (Seuthe et al. 2021). Increasing the number of trials in future studies could provide more stable estimates of *time to APA*. More trials could also allow us to study across trial variability, a factor that has been associated with PD in some, but not all, studies (De Waele et al. 2024; Lin et al. 2016; Roemmich et al. 2012). Nonetheless, among the patients with PD, the *time to APA* was relatively strongly associated (r_s_ = 0.654**;** *p* < 0.001) with the Timed Up and Go scores, a test previously associated with cognitive function and fall risk (Çekok et al. 2020; Mirelman et al 2014). Still, the magnitude of the correlation indicates that other factors are needed to fully explain the variance of the *time to APA* and/or that physiologic noise due to the small number of repetitions may have been a factor. Additionally, participants with PD were tested in their ON medication state, meaning that our findings reflect the influence of dopaminergic therapy. Future research should investigate *time to APA* in the OFF state to determine whether it is a stable marker of disease severity.

We note, too, that we examined upper limb and lower limb reaction times separately. In the future, it would be interesting to explore the neural mechanisms linking motor planning across different effectors. Methods such as EEG and EMG, the simultaneous study of medial–lateral stability, and the assessment of a lower extremity SRT and CRT could also provide further insight into the cortical dynamics underlying *time to APA* (Jacob & Nora 2022; Watanabe & Higuchi 2022). In addition, it is important to keep in mind that while several meaningful and statistically significant associations were observed, the relatively small sample size, especially within each group, may limit the ability to detect more subtle differences or effects. Without an a prior power analysis, it is difficult to definitively state whether the power was fully satisfactory. Future studies should examine these questions using larger sample sizes. We also point out that we examined associations using correlations; an alternative approach is to use mixed-effect models or regression analyses. We opted to use correlation analyses as this does not make any assumptions about cause-and-effect and the direction of the associations; however, this may have come with some negative costs. Our choice of using a random delay, rather than a fixed delay to signal the cue to start, also has implications since some studies have indicated that APA duration is longer in the random delay condition in people with PD (Lu et al. 2017). In the future, it will be interesting to compare *time to APA* in fixed and random delays. Finally, as mentioned above, we note that the present study focused on patients with PD who did not experience FOG. Given the importance of start hesitation to FOG and the previously reported differences in APA characteristics in those PD patients who have FOG, as compared to those who do not (Palmisano et al. 2022; Lencioni et al. 2024; de Lima-Pardini et al. 2020), it would also be interesting to examine *time to APA* in patients with PD and FOG.

## Conclusions

Our findings indicate that *time to APA* is a cognitively demanding process, engaging executive function and visuospatial processing, especially in older adults and people with PD. Its association with both motor efficiency and cognitive performance suggests potential as a biomarker for gait-related cognitive impairment. A shorter *time to APA* appears to enhance motor efficiency and gait performance, particularly in PD. Future research should further explore the neural mechanisms underlying this process and its predictive value for mobility decline in aging and PD.

## Supplementary Information

Below is the link to the electronic supplementary material.

Supplementary Material 1

## Data Availability

Data will be made available upon reasonable, written request for research purposes and in accordance with ethics committee permissions.
