# Supplementary material for: Motor planning stage of gait initiation: effects of aging, Parkinson’s disease, and associations with cognitive function
Source: Exp Brain Res. 2025 Aug 28;243(9):201. doi: 10.1007/s00221-025-07151-3 (PMC12394281; doi:10.1007/s00221-025-07151-3)

| Supplementary Information Table 1: **Results of mixed effects analyses for within subject analyses. p-values of within-group reaction time and accuracy comparison** | | | | |
| --- | --- | --- | --- | --- |
|  | **Reaction time** | | | **CRT Accuracy** |
|  | **SRT vs CRT** | **time to APA vs SRT** | **time to APA vs CRT** | **On vs Off** |
| Young Adults | <0.001* | <0.001* | <0.001* | 0.009* |
| Older Adults | <0.001* | <0.001* | 0.781 | <0.001* |
| People with Parkinson’s disease | <0.001* | <0.001* | 0.107 | <0.001* |

*Significant after a Bonferroni correction. Entries are unadjusted p-values. See Supplementary Table 3 for the values of these measures. 'On' and 'Off' refer to the instructions for the CRT task (i.e., when the lights on the stick were not illuminated, Off, or were illuminated, On).

| Supplementary Information Table 2: **Mixed effect analyses across group results. p-values of between-group reaction time and accuracy comparisons** | | | |
| --- | --- | --- | --- |
|  | **Young adults vs Older Adults** | **Young Adults vs People with PD** | **Older Adults vs People with PD** |
| **SRT** | 0.014 | 0.001* | 0.749 |
| **CRT** | 0.145 | 0.001* | 0.112 |
| **Time to APA** | 0.056 | 0.002* | 0.215 |
| **On accuracy** | <0.001* | 0.011* | 0.098 |
| **Off accuracy** | <0.001* | <0.001* | 0.839 |
| **All accuracy** | <0.001* | <0.001* | 0.249 |

*Significant after a Bonferroni correction. Entries are unadjusted p-values. See Supplementary Table 3 for the values of these measures. On, Off and All refer to the CRT stick task. (i.e., when the lights on the stick were not illuminated, Off, or were illuminated, On).

**Supplementary Information Table 3: Reaction time duration and complex reaction time accuracy rate.**

| People with PD (n=27) | Older adults (n=31) | Young adults (n=34) |  |
| --- | --- | --- | --- |
| 168.7 ± 19.14 *** | 166.7 ± 20.4****** | 152.5 ± 14.5 | SRT (ms) |
| 199.4 ± 27.2*** | 211.7 ± 29.4 | 220.9 ± 24.4 | CRT (ms) |
| 219.9 ± 52.21*** | 208.6 ± 49.8 | 182.2 ± 21.5 | Time to APA (ms) |
| 79.16 ± 15.37*** | 71.03 ± 16.81** | 89.71 ± 10.99 | CRT On accuracy (%) |
| 40.97 ± 23.59*** | 44.78 ± 22.53** | 78.46 ± 15.72 | CRT Off accuracy (%) |
| 58.86 ± 13.49*** | 56.58 ± 13.65** | 84.21 ± 9.91 | CRT All accuracy (%) |

**significant difference between young adults and older adults; ***significant difference between young adults and people with PD.  Data presented as Mean ± SD or as a percentage of correct responses. CRT On and Off refer to when the lights on the stick were not illuminated, Off, or were illuminated, On.

**Supplementary Information Figure 1: Time to APA and ReacStick reaction times, cognitive function and mobility measures scatter plots.** YA: young adults. OA: older adults. PD: people with Parkinson’s.


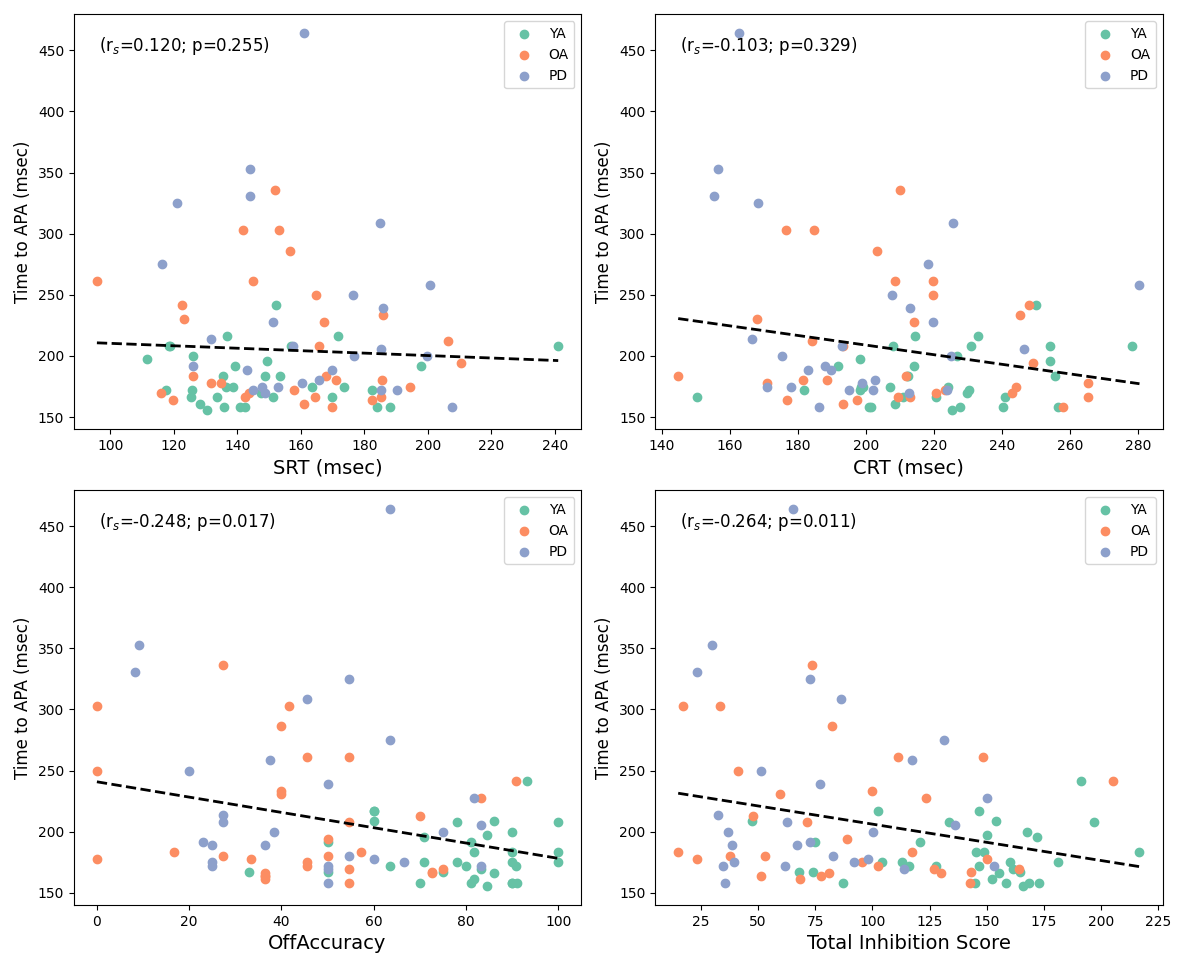

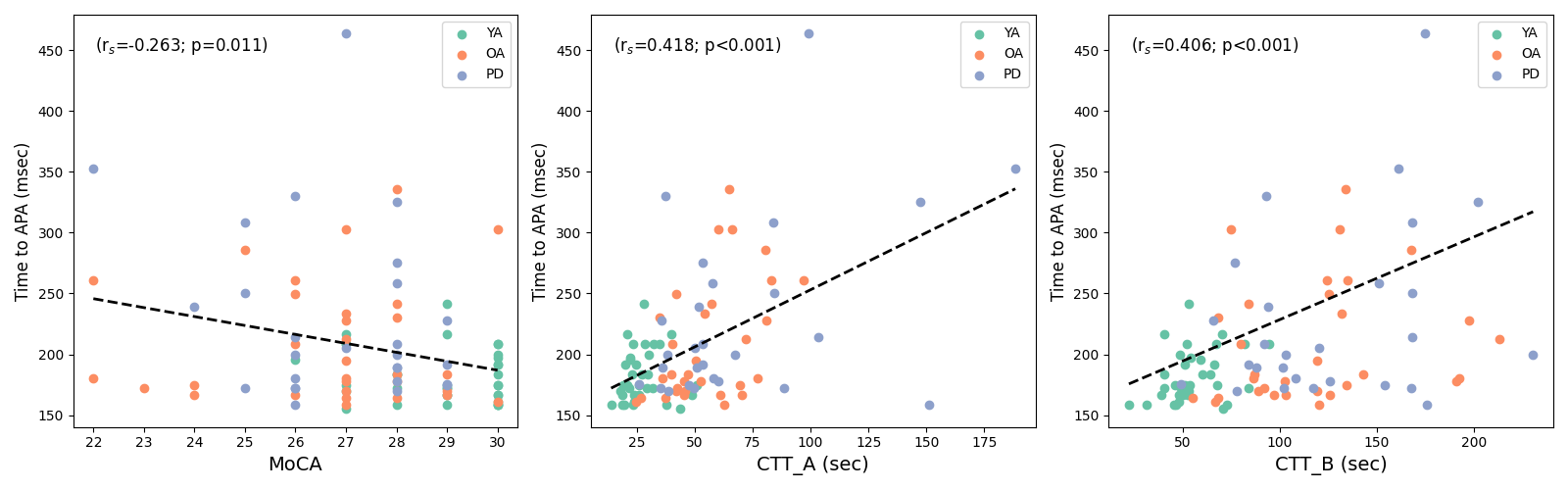

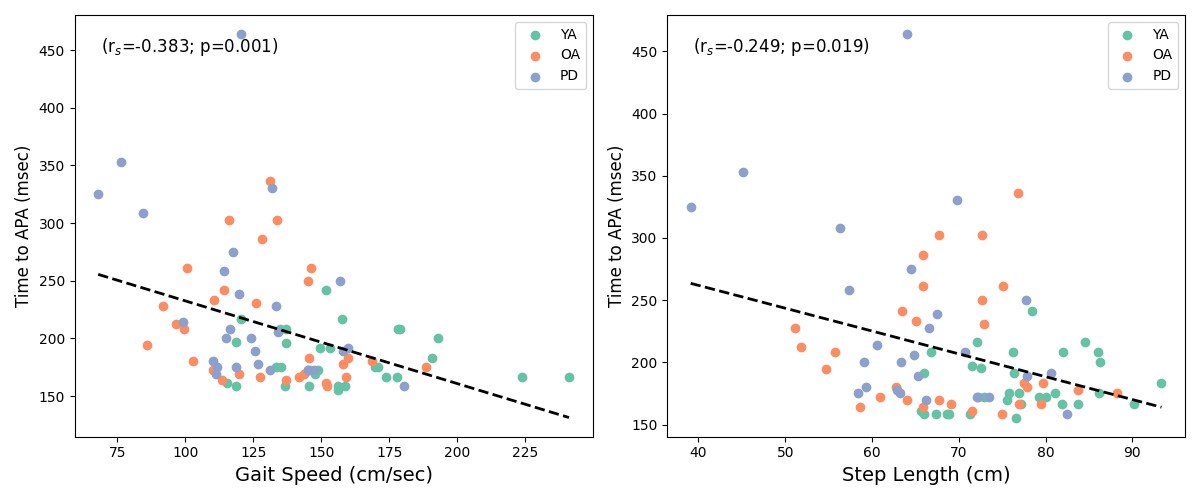

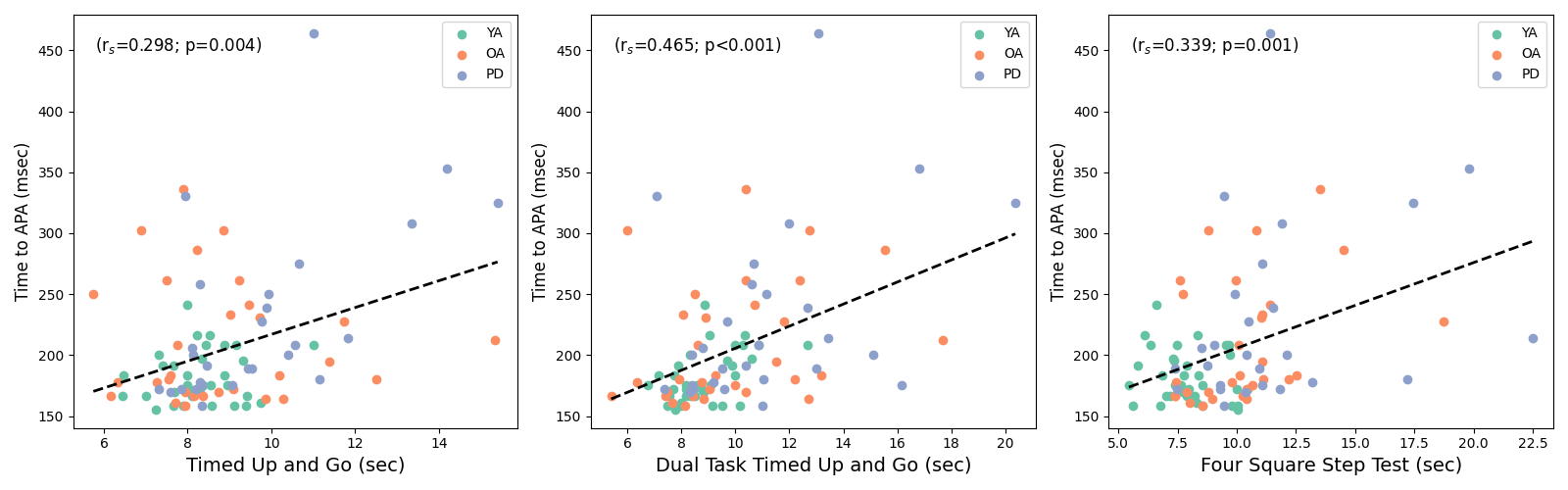

Supplement: Supplementary file 1 — Supplementary Material 1 [file 221_2025_7151_MOESM1_ESM.docx]
